# Increase in methemoglobin fraction due to the use of glyceryl trinitrate patches in preterm infants: a case report and literature review

**DOI:** 10.3389/fped.2025.1505233

**Published:** 2025-02-12

**Authors:** Andrea Calandrino, Francesco Vinci, Marcella Battaglini, Samuele Caruggi, Chiara Andreato, Carolina Montobbio, Marianna Vannati, Diego Minghetti, Giorgia Brigati, Luca Antonio Ramenghi

**Affiliations:** ^1^ Department of Neuroscience, Rehabilitation, Ophthalmology, Genetics, Mother and Child Health, School of Medical and Pharmaceuticals, University of Genoa, Genoa, Italy; ^2^ Neonatal Intensive Care Unit, Department of Maternal and Neonatal Health, IRCCS Istituto Giannina Gaslini, Genoa, Italy

**Keywords:** neonates, prematurity, methemoglobinemia, nigroglycerine, ischemia

## Abstract

**Background:**

Oxidized heme iron forms methemoglobin (MeHb), impairing the oxygen-binding capability of hemoglobin molecules. Nitric oxide (NO) obtained from glyceryl trinitrate (GTN) patches can cause MeHb formation during limb ischemia topical treatment. This case reports a preterm infant treated with multiple GTN patches who developed elevated MeHb levels, a potential therapy complication.

**Case presentation:**

A preterm female newborn (25 + 4 weeks, 560 g) was delivered by cesarean due to maternal HELLP syndrome and intubated for respiratory distress. After developing ischemia in her right hand and foot, GTN patches were applied, but therapy caused a peak in methemoglobin (MeHb) levels, prompting patch removal. MeHb levels normalized after 18 h, and after adjusting therapy, ischemia resolved successfully.

**Discussion:**

We report the case of a premature IUGR infant who developed elevated methaemoglobin (MeHb) levels during topical GTN therapy for catheter-related extremity ischemia. While GTN is effective for neonatal ischemia, its dosing, and safety lack consistent guidelines. Elevated MeHb levels, noted in similar cases here reviewed, can impair oxygen exchange, especially in vulnerable preterm infants with immature skin and reduced enzyme activity.

**Conclusion:**

This case highlights the need for careful MeHb monitoring and a multidisciplinary approach to manage ischemia safely in neonates undergoing GTN therapy.

## 1 Background

The primary role of haemoglobin (Hb) in organisms is to facilitate oxygen transport. This function is ensured by the presence of a reduced form of heme iron (ferrous form, Fe2+) in the Hb structure (1). Methaemoglobin (MeHb) represents an altered structure of Hb caused by the oxidation of the heme iron into the dysfunctional ferric form (Fe3+), which cannot bind oxygen molecules. Inside red blood cells, a system known as the “MeHb reduction system” prevents this phenomenon through the nicotinamide adenine dinucleotide (NADH)-MeHb reductase pathway. This pathway involves a soluble cytochrome b5 and NADH-cytochrome b5 reductase (2). However, when the equilibrium between oxidation and reduction is disrupted by factors such as excessive oxidants, reduced reducing capacity (genetically determined or transient), or abnormal Hb forms, it can lead to methemoglobinemia, which sometimes represents a lifelong concern (3).

Among the oxidants, nitric oxide (NO), an endogenous mediator of essential processes, such as vasodilation and transmission of nerve impulses, plays an important role. NO has a short half-life of approximately 4 s and acts by activating the guanylate cyclase enzyme, thus increasing cyclic guanosine monophosphate (cGMP) levels, which causes vasodilatation and improves circulation (4). The process includes binding to the heme group of Hb, resulting in the formation of MeHb, followed by the elimination of nitrites and nitrates, primarily through renal excretion (5).

Many topical vasodilators, such as glyceryl trinitrate (GTN) patches, are based on NO to reduce local smooth muscle tone (6). Once absorbed by the skin barrier, this substance can gradually release NO in a relatively safe amount, even if the reaction proceeds in a sigmoidal fashion, which makes it relatively easy to reach toxic levels (7).

Severe limb ischemia after arterial catheterization is a known concern in premature infants. Arterial lines are often inserted for blood gas analysis, blood tests, and continuous monitoring of systemic blood pressure, particularly during the first few days after birth. However, this practice can lead to ischemia and tissue necrosis in the extremities, primarily due to the narrow arterial diameter and immature vascular compensatory mechanisms, particularly in infants with very low birth weight (VLBW) and/or intrauterine growth restriction (IUGR) (8).

Topical applications of vasoactive patches have proven to be effective among therapies (9). However, in some cases, they have been linked to systemic complications, primarily due to elevated MeHb levels during treatment, which sometimes necessitate an increase in the administered oxygen fraction (10, 11).

Here, we report the case of an extremely preterm infant who underwent topical therapy with GTN for catheter-related limb ischemia and displayed a similar increase in the MeHb fraction.

## 2 Case presentation

A female newborn was delivered at 25 + 4 weeks of gestational age with a birth weight of 560 g (13th centile according to Bertino 2010 Italian growth charts) (12) by urgent cesarean section for maternal HELLP syndrome (hemolysis, elevated liver enzymes, and low platelets). Pregnancy was achieved by medically assisted procreation and was characterized by severe IUGR with evidence of impaired umbilical and cerebral arterial velocimetry and severe preeclampsia under pharmacological therapy. APGAR scores 0, 6, 8 at 1st, 5th, and 10th minutes respectively. The patient was intubated in the delivery room and mechanically ventilated for the following two weeks of life, initially by conventional pressure-supported ventilation, followed by high-frequency oscillatory ventilation for severe hypercarbia. Due to lung immaturity and elevated FiO2 requirements (100% with SpO2 values persistently below the desired target, despite maximized ventilatory support), two exogenous surfactant administrations were necessary according to the European Consensus Guidelines on the Management of Respiratory Distress Syndrome (13).

Within the first hour of life, an umbilical arterial line was positioned for arterial pressure monitoring. Owing to persistent hypotension, target pressure was achieved using incremental doses of dopamine, dobutamine, and adrenaline. Inotropic therapy was required for a week and then discontinued.

**Figure 1:**
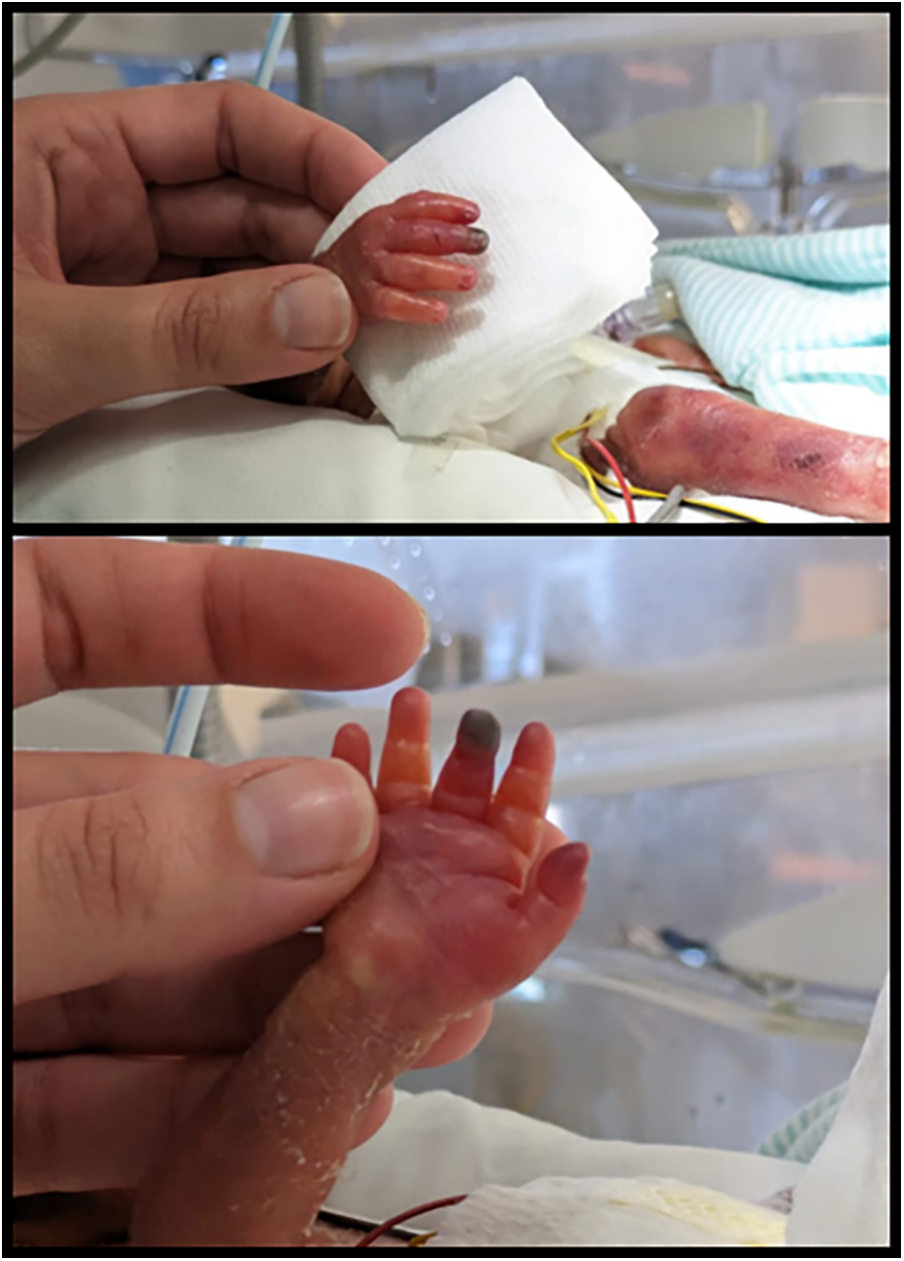
Right-hand ischemia of the 1st, 2nd, and 3rd fingers before GTN patch application.

After 5 h of life, the patient displayed impaired perfusion in the right hand's 1st, 2nd, and 3rd fingers (Figure 1). GTN topical therapy was promptly initiated by applying a half patch on the site (the full patch is 9 cm^2^ containing 18.7 mg of the drug) (14). After 16 h of life, similar signs of ischemia were noticed in the homolateral foot, so an additional half of a GTN patch was applied.

During routine blood gas analyses, a severe consensual peak of 5.9% (determined at 18 h of life) in the MeHb% fraction was recorded, so both patches were promptly removed. After another 18 h, a new determination confirmed the normalization of MeHb% levels; a new application of a ¼ patch was performed at both ischemic sites.

Therapy was continued at the same dosage for the following 118 h and then discontinuation was attempted with success and total recovery of tissue perfusion.

We were able to rule out the existence of congenital methemoglobinemia since MeHb% levels became normal after GTN therapy discontinuation.

**Figure 2:**
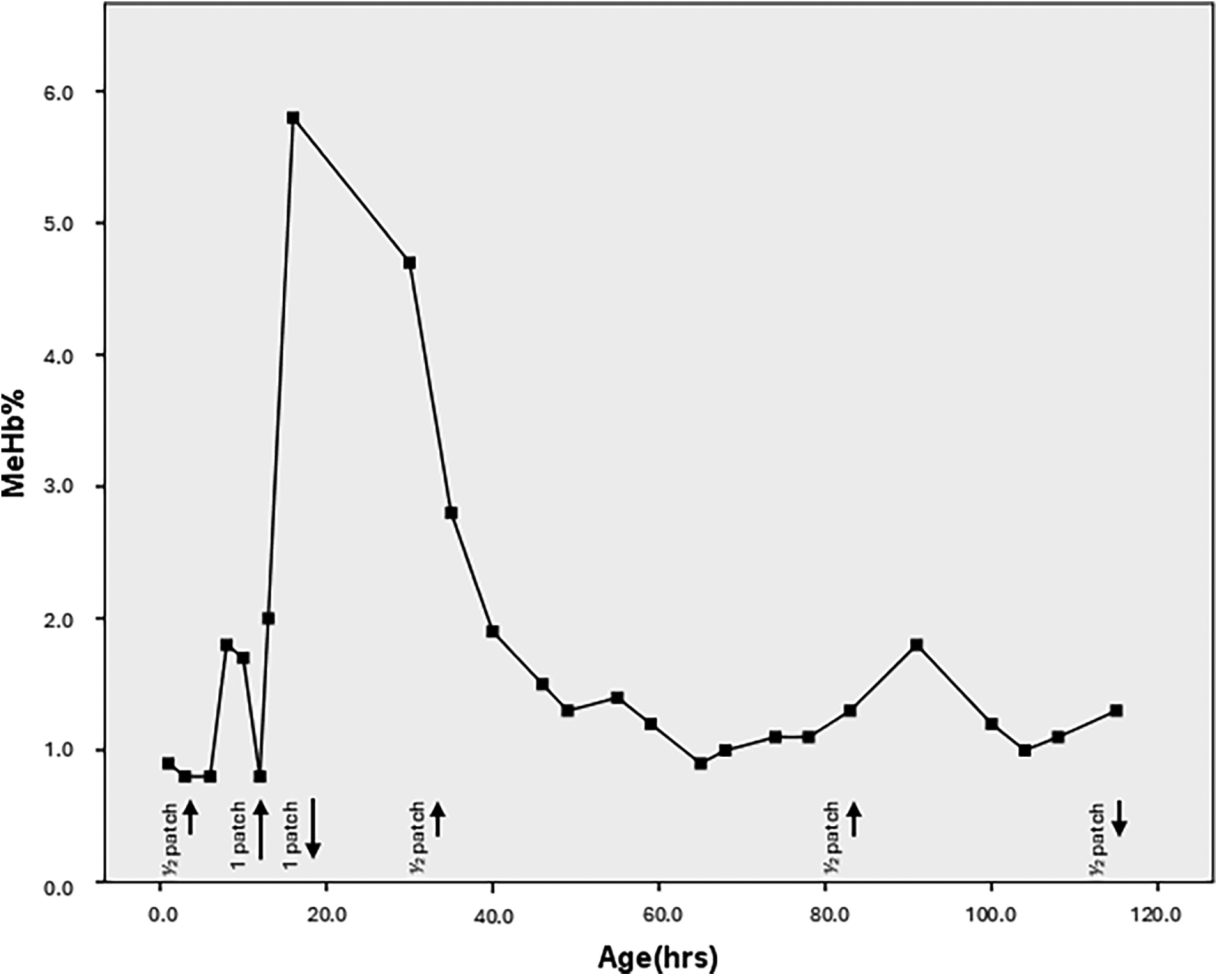
Trend of the meHb% levels according to the hours of life. The black arrows indicate the application of the GTN patches. Short lines represent the use of half a patch, while long lines represent the application of half a patch at two different sites (totaling one full patch). The upward arrows mark the start of therapy, and the downward arrows indicate when therapy is discontinued. The last three short arrows correspond to the application of a quarter patch at two different sites (totaling half a patch).

Figure 2 reports the entire MeHb% monitoring in detail. It shows a slight oscillatory trend after the application of the additional GTN patch in the right foot (within the safety ranges) and a second minor peak at 8 h after reduction of dose to ¼ patch.

## 3 Discussion

Peripheral ischemia in neonates, particularly in extremely preterm and IUGR infants, is a challenging condition that requires careful management. In these cases, therapies such as GTN, a vasodilator, are commonly employed to improve blood flow, especially when standard conservative treatments fail (9). However, as this case report highlights, GTN therapy can sometimes lead to complications, including a rise in MeHb levels, which may impact oxygenation (11).

MeHb levels are routinely measured via spectrophotometry through point-of-care blood gas analyzers (15), with a reported safety range of less than 1.5% (16, 17). In our case, we observed increased MeHb% levels during topical GTN therapy for arterial catheter-related ischemia. Although this phenomenon has been reported in similar cases, there is limited evidence regarding the exact safety guidelines for using GTN in neonates. The frequency, duration, and safety of GTN use are poorly defined, and this treatment approach remains somewhat controversial (18). A literature review conducted by us identifies only two comparable case reports documenting an elevation in MeHb% levels following topical treatment for catheter-related hypoperfusion in preterm infants (10, 11) (Table 1). We acknowledge another case report, published by Ya Seul Han et al. in 2024, dealing with a highly preterm baby who developed severe tissue ischemia after arterial catheterization of the radial artery and was successfully treated using extensive humidification and topical nitroglycerin ointment application until complete clinical recovery; this article was excluded from our review since it is written in Chinese (19).

**Table 1:** Review of the existing reports.

Article	Patients	GA/BW	Application sites	GTN dose	Arterial catheters	MeHb% max	Duration of therapy	Perfusion outcome
Mintoft et al., 2018 (11)	Case #1	24 wks/650 g	Foot bilateral (2 patches), hand unilateral (1 patch)	56.1 mg	RAL	8.4	2 days	Recovery
	Case #2	24 wks/617 g	Foot unilateral (2 patches)	37.4 mg	TAL	23.3	5 days	Finger necrosis (no amputation required)
Pereira at al., 2022 (10)	Case #3	26 wks/580 g (IUGR)	Foot unilateral (1.5 patches^a^)	28.05 mg	UAL	44.1	1.5 days	Recovery

GA, gestational age; BW, birth weight; GTN, glyceryl trinitrate; MeHb%, percentual fraction of methaemoglobin; RAL, radial artery line; TAL, tibial artery line; UAL, umbilical artery line.

^a^ Erroneous application of 3 full patches for a period of 5 h.

In these cases, the increase in MeHb% levels resulted in a significant and persistent decrease in oxygen saturation, which required increased the fraction of inspired oxygen (FiO2) during mechanical ventilation. However, in our case, it was difficult to establish a direct causal link between elevated MeHb% and increased FiO2, as our patient was extremely preterm and IUGR with unstable respiratory status due to respiratory distress syndrome (RDS) and suspected pulmonary hypoplasia. This differs from the other cases where oxygen requirements were stable before the rise in MeHb% levels, suggesting that their respiratory condition remained more stable and that fluctuations in oxygen needs were likely related to the increase in MeHb%. Therefore, we recommend that FiO2% should not be used as a reliable marker of MeHb intoxication, particularly in critically ill patients.

The reported cases also highlighted a range of MeHb% levels, with peaks between 8.4% and 44.1%. This suggests that even modest increases in MeHb% can impair oxygen exchange, making it critical to monitor infants closely during topical vasodilatory therapy. This is particularly true for extremely preterm and IUGR infants, whose skin is more permeable compared to term-born infants, potentially leading to greater absorption of the drug and an increased risk of adverse effects (20). Additionally, premature infants have lower levels of NADH-MeHb reductase and NADH-cytochrome b5 reductase (21), making them more susceptible to MeHb toxicity. These compensatory mechanisms are reliant on NADH levels, and critically ill infants may face heightened vulnerability due to systemic inflammatory insults compared to those in a more stable postnatal period.

Further complicating the issue is the fact that preterm infants have higher levels of fetal hemoglobin (FHb), which is more easily converted to MeHb than adult hemoglobin (22). This, combined with oxidative stress and reduced co-enzyme levels, makes them more susceptible to MeHb intoxication, as reported in studies on retinopathy of prematurity (23, 24).

While GTN therapy is effective in treating peripheral ischemia in neonates, when other treatments fail (25), its use must be approached cautiously. Several alternative therapies may also offer potential benefits. For example, vasodilatory treatments such as eNOS (endothelial nitric oxide synthase) modulators and prostacyclin analogs could improve peripheral circulation but require careful administration to avoid systemic hypotension (26). Another potential treatment is nimodipine, a calcium channel blocker that effectively reduces vasoconstriction and improves blood flow in neonates with cerebral vasospasm. This drug could also benefit neonates with peripheral ischemia, though further research is needed (27). Emerging therapies, such as stem cell treatments, are also being explored. For instance, human-induced pluripotent stem-cell-derived smooth muscle cells have shown promise in increasing angiogenesis in animal models. Still, their safety and feasibility in neonates, especially in preterm infants, remain uncertain (28).

Surgical interventions like vascular reconstruction or endovascular procedures may sometimes be considered. However, these are typically reserved for more severe cases due to the high risks and technical complexity associated with preterm infants (29).

While several therapeutic options exist for managing peripheral ischemia in neonates, particularly in preterm and IUGR infants, each option comes with its own set of challenges and risks. The management approach must be individualized, taking into account the fragile cardiovascular system, limited capacity for tissue regeneration, and heightened risk of complications in these vulnerable infants. A systematic review has suggested that topical nitroglycerine ointment may offer a favorable balance between effectiveness and safety for treating ischemia in newborns compared to other forms of GTN administration, such as patches or sprays (30). Nonetheless, more robust evidence and clearer guidelines are needed to optimize the use of vasodilators like GTN in this patient population.

## 4 Conclusion

This is the fourth case of increased MeHb during topical vasodilatory therapy for extremity ischemia in an extremely premature baby. The data indicate that an elevated percentage of MeHb is often linked to significant complications, particularly those affecting respiratory and oxidative functions, which can result in severe outcomes in this vulnerable patient population.

Therefore, managing peripheral ischemia in neonates with GTN should involve a multidisciplinary approach that includes close monitoring of blood pressure and MeHb levels, along with comprehensive follow-up examinations.

## Data Availability

The raw data supporting the conclusions of this article will be made available by the authors, without undue reservation.

## References

[1] Mansouri A. Methemoglobinemia. Am J Med Sci. (1985) 289(5):200–9. 10.1097/00000441-198505000-00004 4003427

[2] Yip L Spyker DA. NADH-methemoglobin reductase activity: adult versus child. Clin Toxicol. (2018) 56(9):866–8. 10.1080/15563650.2018.1444768 29488404

[3] Jaffé ER. Methemoglobin pathophysiology. Prog Clin Biol Res. (1981) 51:133–51. 7022466

[4] Ignarro LJ Cirino G Casini A Napoli C. Nitric oxide as a signaling molecule in the vascular system: an overview. J Cardiovasc Pharmacol. (1999) 34(6):879–86. 10.1097/00005344-199912000-00016 10598133

[5] Clark DG Litchfield MH. Role of inorganic nitrite in methaemoglobin formation after nitrate ester administration to the rat. Br J Pharmacol. (1973) 48(1):162–8. 10.1111/j.1476-5381.1973.tb08235.x 4198921 PMC1776100

[6] Chalupský K Bartík P Eklová S Entlicher G. Possible metabolic pathways of conversion of formaldoxime and glyceryl trinitrate to NO. Gen Physiol Biophys. (2003) 22(2):233–42. 14661735

[7] Tarburton JP Metcalf WK. Kinetics of amyl nitrite-induced hemoglobin oxidation in cord and adult blood. Toxicology. (1985) 36(1):15–21. 10.1016/0300-483X(85)90003-4 2862720

[8] Deindl P Waldhör T Unterasinger L Berger A Keck M. Arterial catheterisation in neonates can result in severe ischaemic complications but does not impair long-term extremity function. Acta Paediatr. (2018) 107(2):240–8. 10.1111/apa.14100 28960442

[9] Boyce L Dhukaram V. Transdermal glyceryl trinitrate in the treatment of ischemia following toe deformity correction: a case series. Foot Ankle Int. (2014) 35(11):1226–30. 10.1177/1071100714546887 25125514

[10] Pereira SS Yeldo B Aladangady N. Methaemoglobinaemia associated with use of glyceryl trinitrate patches in an extremely preterm infant. J Paediatr Child Health. (2022) 58(10):1862–3. 10.1111/jpc.15996 35474378 PMC9790526

[11] Mintoft A Williams E Harris C Kennea N Greenough A. Methemoglobinemia during the use of glyceryl trinitrate patches in neonates: two case reports. AJP Rep. (2018) 8(4):e227–9. 10.1055/s-0038-1669945 30345159 PMC6188887

[12] Bertino E Neonatal anthropometric charts: the Italian neonatal study compared with other European studies. J Pediatr Gastroenterol Nutr. (2010) 51(3):353–61. 10.1097/MPG.0b013e3181da213e 20601901

[13] Sweet DG European consensus guidelines on the management of respiratory distress syndrome: 2022 update. Neonatology. (2023) 120(1):3–23. 10.1159/000528914 36863329 PMC10064400

[14] Varughese M Koh THHG. Successful use of topical nitroglycerine in ischaemia associated with umbilical arterial line in a neonate. J Perinatol. (2001) 21(8):556–8. 10.1038/sj.jp.7210567 11774020

[15] Calandrino A Optimizing haemoglobin measurements in VLBW newborns: insights from a comparative retrospective study. Early Hum Dev. (2024) 190. 10.1016/j.earlhumdev.2024.105949 38290276

[16] Kravitz H Elegant LD Kaiser E Kagan BM. Methemoglobin values in premature and mature infants and children. AMA J Dis Child. (1956) 91(1):1–5. 10.1001/archpedi.1956.02060020003001 13275111

[17] Haymond S Cariappa R Eby CS Scott MG. Laboratory assessment of oxygenation in methemoglobinemia. Clin Chem. (2005) 51(2):434–44. 10.1373/clinchem.2004.035154 15514101

[18] Mosalli R Khayyat W Al Qarni S Al Matrafi A El Baz M Paes B. Topical nitroglycerin in newborns with ischemic injuries: a systematic review. Saudi Pharm J. (2021) 29(7):764–74. 10.1016/j.jsps.2021.05.008 34400871 PMC8347849

[19] Han YS Song S Sung TJ Chun J. Successful management of severe peripheral tissue ischemia after arterial catheterization in micro preemies using humidification & topical nitroglycerin. Neonatal Med. (2017) 24(4):197–201. 10.5385/nm.2017.24.4.197

[20] Pinsker JE McBayne K Edwards M Jensen K Crudo DF Bauer AJ. Transient hypothyroidism in premature infants after short-term topical iodine exposure: an avoidable risk? Pediatr Neonatol. (2013) 54(2):128–31. 10.1016/j.pedneo.2012.10.005 23590958

[21] Hamon I Gauthier-Moulinier H Grelet-Dessioux E Storme L Fresson J Hascoet JM. Methaemoglobinaemia risk factors with inhaled nitric oxide therapy in newborn infants. Acta Paediatr. (2010) 99(10):1467–73. 10.1111/j.1651-2227.2010.01854.x 20456277

[22] Fossen Johnson S. Methemoglobinemia: infants at risk. Curr Probl Pediatr Adolesc Health Care. (2019) 49(3):57–67. 10.1016/J.CPPEDS.2019.03.002 30956100

[23] Chan E Liu G-S Dusting G. Redox mechanisms in pathological angiogenesis in the retina: roles for NADPH oxidase. Curr Pharm Des. (2015) 21(41):5988–98. 10.2174/1381612821666151029111127 26510439

[24] Elizabeth Hartnett M. The effects of oxygen stresses on the development of features of severe retinopathy of prematurity: knowledge from the 50/10 OIR model. Doc Ophthalmol. (2010) 120(1):25–39. 10.1007/s10633-009-9181-x 19639355 PMC3708708

[25] Vasquez P Burd A Mehta R Hiatt M Hegyi T. Resolution of peripheral artery catheter-induced ischemic injury following prolonged treatment with topical nitroglycerin ointment in a newborn: a case report. J Perinatol. (2003) 23(4):348–50. 10.1038/sj.jp.7210870 12774147

[26] Carroll J Rao R Steinhorn RH. Targeted therapies for neonatal pulmonary hypertension: beyond nitric oxide. Clin Perinatol. (2024) 51(1):113–26. 10.1016/j.clp.2023.11.008 38325937

[27] McGowan B Khaira G Coghlan MA Shaibani A Alden TD Pardo AC. Use of nimodipine in a neonate with cerebral vasospasm with delayed ischemia from subarachnoid hemorrhage in the posterior Fossa. Pediatr Neurol. (2020) 111:44–5. 10.1016/j.pediatrneurol.2020.06.018 32951659

[28] Gao X Human-induced pluripotent stem-cell-derived smooth muscle cells increase angiogenesis to treat hindlimb ischemia. Cells. (2021) 10(4). 10.3390/CELLS10040792 PMC8066461 33918299

[29] Arshad A McCarthy MJ. Management of limb ischaemia in the neonate and infant. Eur J Vasc Endovasc Surg. (2009) 38(1):61–5. 10.1016/j.ejvs.2009.03.010 19362027

[30] Sushko K Topical nitroglycerin ointment as salvage therapy for peripheral tissue ischemia in newborns: a systematic review. CMAJ Open. (2021) 9(1):E252–60. 10.9778/cmajo.20200129 33731426 PMC8096410

